# Antidiabetic, Antihyperlipidemic, Antioxidant, Anti-inflammatory Activities of Ethanolic Seed Extract of *Annona reticulata* L. in Streptozotocin Induced Diabetic Rats

**DOI:** 10.3389/fendo.2019.00716

**Published:** 2019-10-23

**Authors:** Wenbin Wen, Yukiat Lin, Zhenyu Ti

**Affiliations:** ^1^Department of Nephrology, Heji Hospital Affiliated to Changzhi Medical College, Changzhi, China; ^2^Innoscience Research Sdn Bhd, Subang Jaya, Malaysia; ^3^The Department of General Surgery, Xi'an No. 3 Hospital, Xi'an, China

**Keywords:** *Annona reticulata*, kidney, liver, pancreas, oxidative stress, inflammation

## Abstract

*Annona reticulata* L. (Bullock's heart) is a pantropic tree commonly known as custard apple, which is used therapeutically for a variety of maladies. The present research was carried out to evaluate the possible protective effects of *Annona reticulata* L. (*A. reticulata*) ethanolic seed extract on an experimentally induced type 2 diabetes rat model. Male Albino Wistar rats were randomly divided into five groups with six animals in each group viz., control rats in group I, diabetic rats in group II, diabetic rats with 50 and 100 mg/kg/bw of ethanolic seed extract of *A. reticulata* in groups III and IV, respectively, and diabetic rats with metformin in group V. Treatment was given for 42 consecutive days through oral route by oro-gastric gavage. Administration of *A. reticulata* seed extract to diabetes rats significantly restored the alterations in the levels of body weight, food and water intake, fasting blood glucose (FBG), insulin levels, insulin sensitivity, HbA1c, HOMA-IR, islet area and insulin positive cells. Furthermore, *A. reticulata* significantly decreased the levels of triglycerides, cholesterol, LDL, and significantly increased the HDL in diabetic rats. *A. reticulata* effectively ameliorated the enzymatic (ALT, AST, ALP, GGT) and modification of histopathological changes in diabetic rats. The serum levels of the BUN, creatinine levels, uric acid, urine volume, and urinary protein were significantly declined with a significant elevation in CCr in diabetic rats treated with *A. reticulata*. MDA and NO levels were significantly reduced with an enhancement in SOD, CAT, and GPx antioxidant enzyme activities in the kidney, liver, and pancreas of diabetic rats treated with *A. reticulata*. Diabetic rats treated with *A. reticulata* have shown up-regulation in mRNA expression levels of nuclear factor erythroid 2-related factor 2 (Nrf2), NAD(P)H:quinone oxidoreductase 1 (NQO1), Heme oxygenase-1 (HO-1) and protein expression level of Nrf2 with diminution in Keap1 mRNA expression level in pancreas, kidney, and liver. From the outcome of the current results, it can be inferred that seed extract of *A. reticulata* exhibits a protective effect in diabetic rats through its anti-diabetic, anti-hyperlipidemic, antioxidant and anti-inflammatory effects and could be considered as a promising treatment therapy in the treatment of diabetes mellitus.

## Introduction

Diabetes mellitus (DM) is a metabolic disorder characterized by the presence of high levels of glucose in blood that occurs either due to insulin's deficiency or malfunction (1, 2). Generally, people with diabetic condition encounter uncountable misery and devastating complications that may lead to morbidity and mortality (3). As per recent estimates by International Diabetes Federation, 5 million deaths were recorded due to diabetes (3). Earlier, it has also been reported that more than 2.5% of the world's total population suffer from DM (4). This increased prevalence rate, along with debilitating complications, warrants an urgent need to search for effective treatment strategies. Hyperglycemia-induced oxidative stress in terms of an increased generation of reactive oxygen/nitrogen species (ROS/RNS) and suppression of antioxidant defenses, such as SOD, CAT, GPx, play vital roles in the pathogenesis of DM (5, 6). This loss of balance in ROS/RNS and antioxidant defense mechanism may make tissues more vulnerable to oxidative stress that further aggravates the complications of DM. To negate the oxidative stress, cells are equipped with redox sensitive transcription factor Nrf2 to provide cellular protection (7). Earlier, it has also been reported that cellular redox homeostasis occurs primarily at the transcriptional level through the regulation of Nrf2/Keap1/ARE pathway (8, 9). It not only acts as a critical upstream regulator of global antioxidant response but also regulates the genes involved in inflammatory reaction (8, 10). Previously, it was reported that sustained oxidative stress through suppression of Nrf2 signaling pathway directs the overproduction of pro-inflammatory cytokines and chemokines, which activates the transcription factor NF-κβ that leads to chronic inflammation (11). Earlier, Pedruzzi et al. (12) reported that impairment of Nrf2 and its regulated genes results in systemic overload of oxidative stress and inflammation. Hence, the Nrf2/Keap1/ARE pathway represents an essential target to treat a broad spectrum of oxidative stress-mediated diabetic complications (8).

In addition to hyperglycemia-induced oxidative stress and inflammation, hyperlipidemia is another common feature that further progresses the diabetic complications (13). Further, the deterioration of liver and kidney functionality was evident from alterations in liver and kidney enzymatic and functional markers with deteriorated morphology in diabetic rats (14, 15).

Among the various treatment strategies, diet therapy, pharmacotherapy, and insulin therapy are the main treatment options available to control diabetes, in addition to wide range of glucose lowering drugs which exert their hypoglycemic effects through various mechanisms (16). However, these treatment options have not gained much significance as these treatment strategies are commonly associated with disadvantages, such as drug resistance, side effects, and toxicity. Hence, regardless of the presence of these hypoglycemic pharmacological drugs, supplementation of herbal based drugs to treat DM is now a promising and novel treatment strategy due to its safe and non-toxic nature (17).

Earlier, various herbal medicinal plants and herbs, such as *Averrhoa bilimbi* Linn. (18), *Potentilla discolor* Bunge (19), and *Semecarpus anacardium* Linn., which contain considerable amounts of antioxidant and anti-lipidemic components, have been found to be helpful in the management of DM and its associated complications. Considering the antioxidant and anti-lipidemic protective principles from herbal medicinal plants to fight DM, the present study is focused on *Annona reticulata* L.

The *Annona reticulata* belongs to the family Annonaceae and more than 100 different species of Annona genus have been identified (20). It is a traditional plant, commonly known as Bullock's heart and it has been used to treat various disorders such as epilepsy, cardiac problems, dysentery, worm infestations, bacterial infections, hemorrhage, dysuria, fever, and ulcers (21). The locals of Philippines, India and some other countries have claimed that this plant was traditionally used as anti-inflammatory, anti-stress, and anti-helminthic medications (22). Therefore, we chose to study the anti-inflammatory properties using a diabetic rat model to relate the pharmacological significance with the ethnobotanical claims by locals. Further, studies carried out using extracts of different parts of the plant have been reported to have anti-cancer (23), anti-inflammatory (24), anti-oxidant (25), hypoglycemic (26), analgesic (27), and anti-ulcerative effects (28), as well as wound healing activity (29).

Considering the diabetes-mediated complications and protective principles of *A. reticulata*, this study was performed to determine the anti-diabetic, anti-hyperlipidemic, antioxidant, and anti-inflammatory actions of *A. reticulata* seed in diabetic rats. The study also broadened its scope by studying the role of Nrf2/Keap1 molecular pathway in modifying the effects of *A. reticulata*.

## Materials and Methods

### Chemicals

Streptozotocin was obtained from Sigma Chemicals, St. Louis, MO, USA. ELISA kits (NF-kβ, IL-1β, and IL-6) were procured from Abcam, UK. All other chemicals and solvents used were of analytical grade and procured from Sigma Chemicals, St. Louis, MO, USA. All primary antibodies used in this study were purchased from Santa Cruz Biotechnology, USA.

### Preparation of Extract

The plant materials were bought from a local market and rinsed with tap water to clean from extraneous materials. The seeds of *A. reticulata* were dried under shade at room temperature, crushed by a mechanical grinder and were sieved through 40 mesh. The pounded materials were extracted with ethanol (95%) using Soxhlet extraction apparatus. The extract was concerted under reduced pressure. Thus, the ethanol free semi-solid mass gained was used for further studies.

### Gas Chromatography-Mass Spectrometry (GC-MS) Analysis

GC-MS analysis of the seeds of *A. reticulata* was performed using a GC-MS-QP2010 (Shimadzu, Japan), comprising of an AOC-20s headspace sampler and an AOC-20i autoinjector with a MS analysis capillary column (30 mm length × 0.25 mm diameter and 0.25 μm film thickness). Injector temperature was 250°C (Split injection mode). The temperature program was as follows: 80°C (3 min) and then with an increase of 10°C/min to 280°C. Carrier gas used was pure (99.999%) Helium (40.5 cm/s linear velocity) and constant column flow was 1.21 ml/min (total flow of 16.3 ml/min). Finally, the components of the seed extract were identified based on GC retention time and MS interpretation by matching spectra with NIST library.

### Experimental Animals

Male healthy rats (150–250 g body weight) of Wistar strain were obtained from Central Animal House of our Institute and used for the study. The animals were housed for a week under standard conditions (12 h dark: 12 h light cycle, 50–60% relative humid and room temperature) before starting the actual experiments. This study was carried out as per recommendations from animal ethical committee of Changzhi Medical College by following the guidelines of National Institute of Health. The animal ethical committee of our institute approved the protocol. The rats were fed with normal rat feed and water *ad libitum*. All the experiments were done at regular time points.

### Induction of Type 2 Diabetes Mellitus

For the induction of type 2 diabetes mellitus, overnight fasted rats were injected with nicotinamide (NA; 110 mg/kg; dissolved in saline) to minimize the streptozotocin (STZ) induced pancreatic β-cell damage. Fifteen minutes later, STZ (55 mg/kg) dissolved in 0.1 M citrate buffer (pH:4.5) injected intraperitoneally (i.p.) for the induction of type 2 diabetes (30). After 7 days of induction, the fasting blood sugar levels (FBG) were determined by glucometer and the rats with FBG level >250 mg/dL were considered to be diabetic and were used in the study.

### Experimental Design

**Figure 1:**
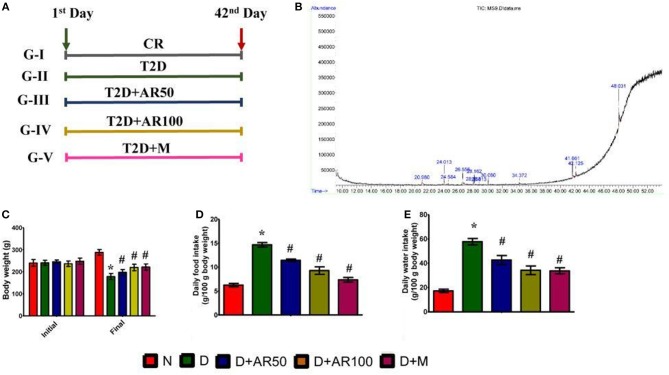
**(A)** scheme of treatment, CR = Control rats; T2D = type 2 diabetic rats; T2D+AR50 = Type 2 diabetic rats + 50 mg/kg bw of *A. reticulata* seed ethanolic extract; T2D+AR100 = Type 2 diabetic rats + 100 mg/kg bw of *A. reticulata* seed ethanolic extract; T2D+M = Type 2 diabetic rats + Metformin; **(B)** GC-MS chromatogram of ethanolic seed extract of *Annona reticulata* L; Effect of *Annona reticulata* L on **(C)** Body weight **(D)** Daily food intake, and **(E)** Daily water intake. Each value is mean ± SD of six rats in each group. ^*^significant compared to normal control group *p* < 0.01. ^#^significant compared to diabetic group *p* < 0.01.

Male Wistar Albino rats were divided into five groups. Rats in group I served as normal controls, while rats in group II-V served as diabetic rats. Diabetic animals in group III and IV were administered orally with 50 and 100 mg/kg bw of *A. reticulata* seed ethanolic extract, respectively, and group V diabetic animals were treated orally with standard drug metformin at the dosage of 1 mg/kg bw for 42 consecutive days. The simplified scheme of treatment is given in Figure 1A.

### Oral Glucose Tolerance Test (OGTT) and Insulin Tolerance Test (ITT)

The OGTT was carried out 1 week before the end of experiment. Overnight fasted rats were administered with glucose (2 g/kg) orally after administration of extract. Blood samples were collected from tail vein at 0, 30, 60, 90, and 120 min after glucose administration. ITT was performed 48 h before the last day of experimental period. Overnight fasted rats were injected intraperitoneally with 0.75 U/kg insulin. The blood glucose levels were estimated at 0, 30, 60, 90, and 120 min after insulin injection.

### Blood Glucose Level and Biochemical Parameters Measurement

After 42 days of experimental period, the animals were sacrificed under chloroform anesthesia. The serum was separated from the collected blood and used for biochemical parameters. Liver, kidney, and pancreas were quickly removed and rinsed with saline. Glucose level was estimated by using Glucometer (One Touch Horizon, Lifescan, Johnson and Johnson Company). Homeostatic model assessment (HOMA)-IR, an indicator of beta cell function and insulin resistance, was done by the method of Matthews et al. (31). HOMA-IR was calculated from the following formula: HOMA-IR = [Fasting glucose (mmol/L) × fasting insulin (mU/L)]/22.5. Insulin was assayed by the solid phase enzyme-linked immunosorbent assay (ELISA). Lipoproteins were fractionated by a dual precipitation technique of Wilson and Spiger (32) and high-density lipoprotein (HDL), low-density lipoprotein. Total cholesterol (TC) and triglycerides (TG) were measured using commercially available kits (Abcam, UK) following manufacturer's protocol. Glycosylated hemoglobin (HbA1C) were estimated by the method of Rao and Pattabiraman (33).

Lipid peroxidation levels were determined in the liver, kidney, and pancreatic tissues spectrophotometrically by measuring the content of thiobarbituric acid reactive product, malondialdehyde (MDA) at 535 nm according to the method of Garcia et al. (34). The results were expressed as nanomole of MDA formed per milligram protein. Superoxide dismutase (SOD) activity was measured by rate of inhibition of pyrogallol auto-oxidation on a spectrophotometer at 470 nm according to the method of Marklund and Marklund (35). SOD activity was expressed as units per milligram protein. Glutathione peroxidase (GPx) activity was estimated by the rate of NADPH oxidization spectrophotometrically at 340 nm following the method of Rotruck et al. (36). GPx activity was expressed as micrograms of reduced glutathione (GSH) consumed/ min/ mg protein. Catalase (CAT) activity was estimated by monitoring the decomposition of H_2_O_2_ spectrophotometrically at 240 nm following the method of Sinha (37). CAT activity was expressed as micromoles of H_2_O_2_ consumed/min/mg protein. Nitric oxide (NO) concentrations were measured using Griess reaction following the manufacturer's protocol (Sigma Aldrich, St. Louis, USA). Sodium nitrite solution was used as a standard to measure the nitrite concentrations and the absorbance was read at 540 nm. Serum enzymes alkaline phosphatase (ALP), aspartate aminotransferase (AST), alanine aminotransferase (ALT), and gamma-glutamyl transferase (GGT) levels were estimated using commercial kits (Stanbio Reagent kit, TX, USA) by following the manufacturer's instructions. Tissue protein concentrations were estimated using a Randox kit with an automated Randox Daytona analyzer (Randox Laboratories Ltd., USA) following protocols from the manufacturer.

Urea was measured by the method of Natelson et al. (38), uric acid was measured by the method of Eichhorn and Rutenberg (39) and creatinine was measured by the method of Owen et al. (40) for the evaluation of kidney damage.

### Quantitative Reverse Transcription Polymerase Chain Reaction (PCR) Analysis

The extraction of total RNA in the samples was performed using a TRIzol® Reagent method, quantified using Nanodrop 2000, and reverse transcription was done with the help of DNA synthesis kit (PE Applied Biosystems, Foster City, CA, USA).

The following list of primers was used the analysis:

Nrf2: Forward - 5′AGCACATCCAGACAGACACCA3′,
Reverse - 5′TATCCAGGGCAAGCGACTC3′;
Keap1: Forward - 5′AGCAGGCTTTTGGCATCAT3′,
Reverse - 5′CCGTGTAGGCGAACTCAATTAG3′;
(NQO-1):Forward - 5′GAGAAGAGCCCTGATTGTACTG3′;
Reverse - 5′ACCTCCCATCCTCTCTTCTT3′;
HO-1):forward - 5′CTCCCTGTGTTTCCTTTCTCTC3′;
Reverse - 5′CTGCTGGTTTCAAAGTTCAG3′;
β-actin:Forward - 5′GGTATCCTGACCCTGAAGTA3′;
Reverse - 5′CACACGCAGCTCATTGTAGA3′.

RT-PCR was performed using the SYBR GREEN PCR master mix using Applied Biosystems Real-Time PCR System. The Relative quantification of mRNA expression was done using the 2^−ΔΔCt^ method. Ct values were normalized by β-actin to compare the expression among different groups.

### Measurement of NF-κB p65, IL-1β, and IL-6

NF-κB p65, IL-1β, and IL-6 concentrations in various tissues of rat, such as pancreas, kidney, and liver, were measured using commercially available kits (as mentioned in Materials section) as per manufacturers' protocols.

### Histological Analysis

The pancreas, liver, and kidney tissues from each group of animals were quickly removed after sacrificing the animals and washed immediately on ice cold saline. Each small portion of tissues was fixed in 10% neutral formalin fixative solution for carrying out histological studies. Tissues were embedded in paraffin after fixation and then solid sections were cut at 5 μm, which were then stained with haematoxylin and eosin. The photomicrographs of histological studies were captured.

### Immunohistochemistry

Immunohistochemistry was carried out on formalin-fixed and paraffin-embedded sections of tissues. These sections were then deparaffinized, hydrated and washed in 0.1 M phosphate buffer saline (PBS). The presence of endogenous peroxidases was neutralized by treatment with H_2_O_2_ in the presence of methanol (Peroxidase acts as blocking solution), which was then washed in tris buffer saline (TBS). The imprecise binding of IgG was blocked with the help of normal goat serum, which was diluted (1:50) in 0.1% bovine serum albumin (BSA) with TBS for at least 30 min. The sections were incubated with the primary antibodies (Insulin, Nrf2, and NF-kβ), which were diluted and then left overnight. The sections were rinsed thrice each for at least 5 min in buffer and then incubated with biotinylated secondary antibodies diluted 1:1000 for a further 30 min, proceeding with washing. After this they were incubated further 30 min with Vectastain ABC kits (Avidin, Biotinylated horse radish peroxidase Complex) and washed for 10 min. Then the substrate, diaminobenzidine tetra hydrochloride (DAB) in distilled water, was added and incubated for 5–10 min. The enzyme reaction was developed. The slides were lightly counterstained by haematoxylin to gain a good morphological identification of cells, and dehydrated by passing through ascending concentrations of alcohol then cleared by xylene. Permanent mounting media was used to put the cover slip. The substrate produces brown color at the immunoreactive sites.

### Statistical Analysis

The results are presented as mean ± standard deviation (S.D). The differences between groups were analyzed using analysis of variance (ANOVA), followed by Bonferroni *posthoc* test. A value of *p* < 0.05 was considered statistically significant. Statistical software IBM SPSS (Version-18.0; Chicago, IL, USA) was used for analysis.

## Results

### Effects of *A. reticulata* on Body Weight, Food, and Water Intake

The presence of bioactive compounds in the ethanolic extract of *A. reticulata* seeds was identified using GC-MS analysis. The GC-MS chromatogram (Figure 1B) expresses 11 peaks and has been identified after comparison of the mass spectra with NIST library (Table 1). The retention time, molecular formula, weight, and the percentage content in the seed extract were given. On the basis of these data, it was determined that 2,3-Dihydrobenzofuran (7.910%), Deconoin acid ethyl ester (14.730%), 2,3-Dimethoxy-succinicacid dimethyl ester (4.021%), 3-Hexadecyne (13.035%), Allo-Aromadendrene (1.970%), Allo-Aromadendrene (6.739%), Megastigmatrienone (1.901%), Ar-turmerone (3.952%), Oleic acid (10.028%), Gentisic acid (8.496%), and 13-Docosenamide (23.190%) were present in the *A. reticulata* seed extract. Among all, 13-Docosenamide was present in abundant amounts and megastigmatrienone was present in low quantity.

**Table 1:** GC–MS data of the different compounds present in ethanolic extract of *Annona reticulata*.

**S. No**	**RT**	**Name of compound**	**Molecular formula**	**MW**	**Peak area %**
1	20.978	2,3-Dihydrobenzofuran	C_8_H_8_O	120	7.910
2	24.015	Deconoin acid, ethyl ester	C_12_H_24_O_2_	200	14.730%
3	24.581	2,3-Dimethoxy-succinicacid dimethyl ester	C_8_H_14_O_6_	206	4.021%
4	26.552	3-Hexadecyne	C_16_H_30_	222	13.035%
5	28.054	Allo-Aromadendrene	C_15_H_24_	204	1.970%
6	28.157	Allo-Aromadendrene	C_15_H_24_	204	6.739%
7	28.814	megastigmatrienone	C_13_H_18_O	190	1.901%
8	30.082	Ar-turmerone	C_15_H_20_O	216	3.952%
9	41.663	Oleic acid	C_18_H_34_O_2_	281	10.028%
	42.123	Gentisic acid	C_7_H_6_O_4_	370	8.496%
10	48.031	13-Docosenamide, (Z)-	C_22_H_43_NO	337	23.190%

Figure 1C is depicting the alterations in body weight of control and experimental rats. In comparison with control rats, there was a significant decrease in the body weights of diabetic rats. On the other hand, treatment of diabetic rats with *A. reticulata* seed extract (50 and 100 mg/kg bw) and metformin caused a significant increase in the body weights when compared to body weights of diabetic rats. Diabetic condition has resulted in a drastic increase in daily food (Figure 1D) and water intake (Figure 1E), which decreased significantly upon treatment with 50 and 100 mg/kg *A. reticulata* seed extract and metformin, when compared to diabetic rats.

### Effects of *A. reticulata* on Insulin Resistance and Pancreatic Functions

**Figure 2:**
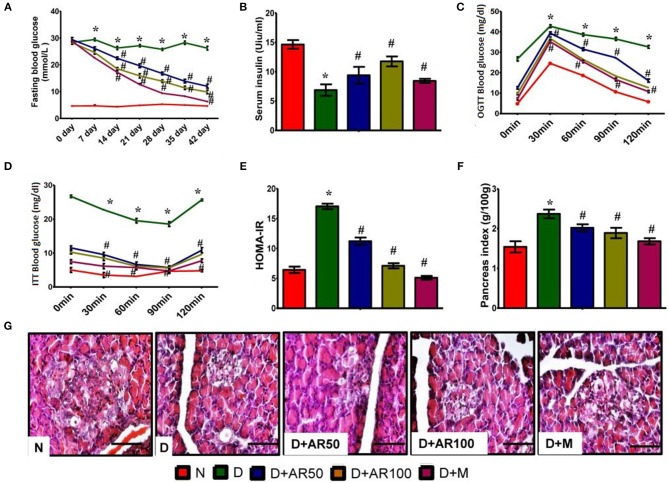
Effect of *Annona reticulata* L on **(A)** fasting blood glucose levels **(B)** serum insulin levels **(C)** Oral Glucose Tolerance Test (OGTT) **(D)** insulin tolerance test (ITT) **(E)** HOMA-IR **(F)** pancreas index **(G)** pancreas histology. Each value is mean ± SD of six rats in each group.^*^significant compared to normal control group *p* < 0.01. ^#^significant compared to diabetic group *p* < 0.01.

There was a significant increase in weekly fasting blood glucose in diabetic rats which did not reduce till 42 days when compared to control rats. Whereas, 50 or 100 mg/kg *A. reticulata* seed extract and metformin treatment for 42 days significantly reduced the fasting glucose levels to near normal levels (Figure 2A).

The prime hallmark of DM i.e., levels of serum insulin were drastically declined in diabetic rats, while *A. reticulata* seed extract or metformin treatment to diabetic rats significantly improved the insulin levels, compared to that of same levels in diabetic controls (Figure 2B).

Figure 2C shows the glucose tolerance as measured by OGTT. Control rats showed an increase in blood glucose levels by 30 min, which reverted to normal levels upon physiological hormonal control. In contrast, diabetic rats showed impaired glucose tolerance and did not revert to normal level. While, *A. reticulata* ethanolic seed extract treatment to diabetic rats significantly improved the glucose tolerance similarly to the standard drug. This finding was further evident by insulin tolerance test as shown in Figure 2D.

Hemoglobin, the oxygen carrier of blood, is glycated by blood glucose and the measurement of glycated hemoglobin (HbA1c) portrays the average blood glucose levels. The value below 6.0 is considered to be normal and above 6.5 is considered to be diabetic. In the present study, diabetic rats showed high HbA1c i.e., above 6.5, while this was lowered on treatment with *A. reticulata* seed extract or metformin. This may be due to the fact that *A. reticulata* seed extract or metformin lowered the blood glucose levels and, thus, the process of glycation is suppressed (Supplementary Figure 1).

The results also showed a drastic increase in HOMA-IR in diabetic rats, indicating the insulin resistance, while this was decreased on treatment with 50 or 100 mg/kg *A. reticulata* seed extract or metformin (Figure 2E).

A significant increase in pancreas index of diabetic rats was observed when compared with normal rats, which resulted in a significant decline in pancreas index upon treatment with either *A. reticulata* ethanolic seed extracts or metformin, compared to diabetic rats (Figure 2F).

Islets of Langerhans are the endocrine patches of pancreas, wherein alpha cells produce glucagon, beta cells produce insulin and delta cells produce somatostatin. Glucagon and insulin play vital roles in glucose homeostasis. The H&E staining of pancreas depicted the morphology of the pancreas, which shows that diabetic rats presented with small Islets of Langerhans with loss of shape, while 50 or 100 mg/kg *A. reticulata* seed extract or metformin treatment restored the shape and size of islet cells (Figure 2G).

The measurement of islet area is important in assessing the anti-diabetic effect of any drug. In the present study, there was a significant decrease in the islet area in diabetic rats, showing the destruction of beta cells that produce insulin. Islet area was significantly increased upon treatment with 50 or 100 mg/kg *A. reticulata* seed extract or metformin (Supplementary Figure 2). IHC of pancreas revealed the presence of less insulin in diabetic rats, while the treatment of diabetic rats with 50 or 100 mg/kg *A. reticulata* seed extract or metformin caused an increase in insulin (Supplementary Figure 3). The results were further supported by the presence of fewer insulin immunopositive sites in diabetic pancreas, however the treatment of 50 or 100 mg/kg *A. reticulata* seed extract or metformin significantly improved the insulin immunopositive sites in diabetic rats (Supplementary Figure 4).

### Effects of *A. reticulata* on Lipid Profiles

**Figure 3:**
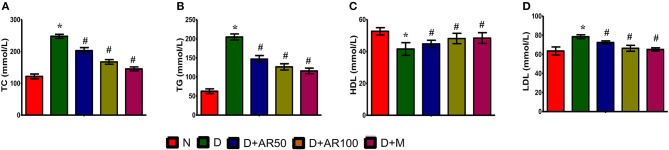
Effect of *Annona reticulata* L on **(A)** Total cholesterol (TC) **(B)** Triglycerides (TG) **(C)** High-density lipoprotein (HDL) and **(D)** Low-density lipoprotein (LDL). Each value is mean ± SD of six rats in each group.^*^significant compared to normal control group *p* < 0.01. ^#^significant compared to diabetic group *p* < 0.01.

Diabetes is often associated with alterations in lipid metabolism and hence lipid profile was studied in the present study. The results showed that significantly increased levels of total cholesterol (Figure 3A), triglycerides (Figure 3B), and LDL (Figure 3C) with significantly decreased levels of HDL (Figure 3D) were observed in diabetic rats compared to control rats. Moreover, 50 or 100 mg/kg *A. reticulata* seed extract or metformin treatment to diabetic rats for 42 days significantly improved the above mentioned lipid metabolism biomarkers.

### Effect of *A. reticulata* on Kidney Histology and Function

**Figure 4:**
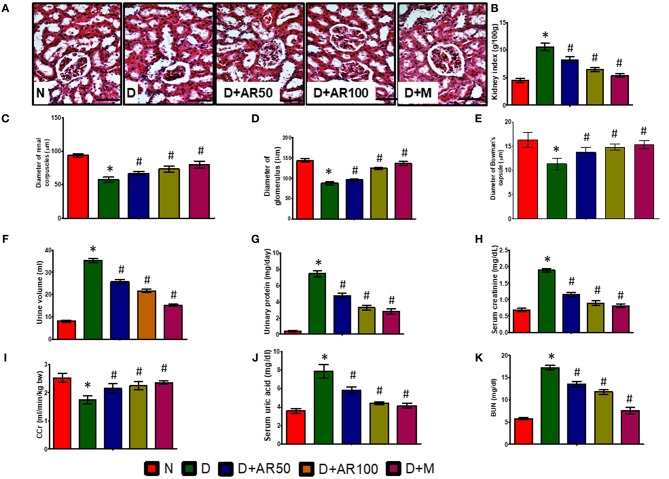
Effect of *Annona reticulata* L on **(A)** Histology of kidney **(B)** kidney tissue somatic index **(C)** Diameter of renal corpuscles **(D)** Diameter of glomerulus **(E)** Diameter of Bowman's capsule **(F)** Urine volume **(G)** Urinary protein **(H)** Serum creatinine **(I)** Creatinine Clearance (CCr) **(J)** Serum uric acid **(K)** Blood urine nitrogen (BUN). Each value is mean ± SD of six rats in each group.^*^significant compared to normal control group *p* < 0.01. ^#^significant compared to diabetic group *p* < 0.01.

Diabetic nephropathy is a common complication associated with kidney as this organ is highly susceptible to high glucose concentrations. As shown in Figure 4A, the histopathological sections of kidney in diabetic rats presented with distorted glomerular morphology, indicating kidney damage. On the other hand, 50 or 100 mg/kg *A. reticulata* seed extract or metformin treated diabetic rats showed an improvement in glomerular injury, in comparison with diabetic rats. The histopathological observations were supported by renal parameters, such as kidney index (Figure 4B), diameters of renal corpuscles (Figure 4C), glomerulus (Figure 4D), and Bowman's capsule (Figure 4E), in terms of deteriorated alterations in diabetic rats, which showed improvement in all these parameters after treatment with either *A. reticulata* seed extract or metformin. Also, urine volume (Figure 4F) and urinary protein excretion (Figure 4G), as a measure of polyuria and proteinuria, respectively, were significantly higher in diabetic rats compared to non-diabetic controls. Whereas, 50 or 100 mg/kg *A. reticulata* seed extract or metformin treatment to diabetic rats significantly decreased the urine volume and urinary protein, compared to diabetic controls. Further, serum creatinine (Figure 4H), uric acid (Figure 4J), and BUN (Figure 4K) were significantly increased with a significant decrease in CCr (Figure 4I) was observed, indicating a kidney incapable of eliminating waste products in diabetic rats compared to that of non-diabetic rats. Treatment of diabetic rats with 50 or 100 mg/kg *A. reticulata* ethanolic seed extract or metformin significantly reduced the serum creatinine, uric acid and BUN with significantly increased CCr, indicating *A. reticulata* seed extract or metformin ameliorated the renal injury.

### Effect of *A. reticulata* on Liver Histology and Function

**Figure 5:**
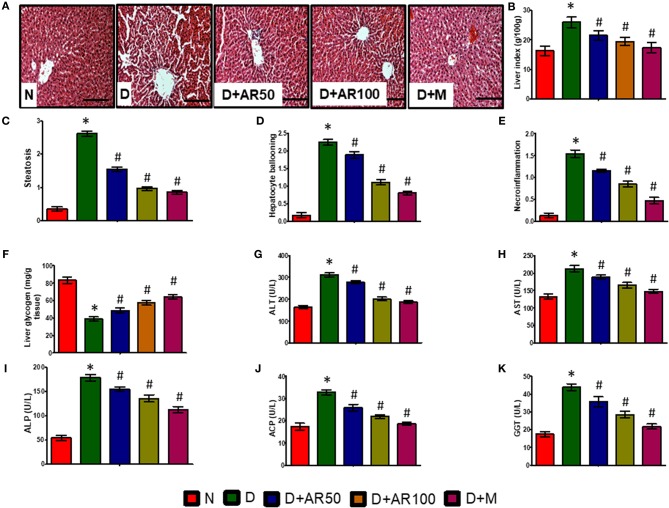
Effect of *Annona reticulata* L on **(A)** Histology of liver **(B)** Liver tissue somatic index **(C)** Steatosis **(D)** Hepatocyte ballooning **(E)** Necroinflammation **(F)** Liver glycogen **(G)** Alanine aminotransferase (ALT) **(H)** Aspartate aminotransferase (AST) **(I)** Alkaline phosphatase (ALP) **(J)** Acid phosphatase (ACP) **(K)** Gamma-glutamyl transferase (GGT). Each value is mean ± SD of six rats in each group. ^*^significant compared to normal control group *p* < 0.01. ^#^significant compared to diabetic group *p* < 0.01.

Liver is the major site of glucose metabolism where glycogenolysis takes place. The histopathological changes of liver in diabetic rats demonstrated distorted morphology with hepatocellular necrosis and vacuolization, while diabetic rats treated with 50 or 100 mg/kg *A. reticulata* seed extract or metformin displayed no major alterations and were near to normal liver structure (Figure 5A). The liver index (Figure 5B), steatosis (Figure 5C), hepatocyte ballooning (Figure 5D), and necroinflammation (Figure 5E) were significantly increased with significantly decreased glycogen content (Figure 5F) in diabetic rats. While, 50 or 100 mg/kg *A. reticulata* and metformin treatment to diabetic rats significantly improved all of these parameters when compared to that of diabetic rats.

The activities of ALT, AST, ALP, ACP, and GGT are considered to be reliable markers to assess liver function. In the present study, serum levels of ALT (Figure 5G), AST (Figure 5H), ALP (Figure 5I), ACP (Figure 5J), and GGT (Figure 5K) were significantly elevated in diabetic rats compared to non-diabetic rats, while diabetic rats treated with 50 or 100 mg/kg *A. reticulata* ethanolic seed extract and metformin for 42 days had much lower serum levels, which markedly alleviated liver damage.

### Effect of *A. reticulata* on Nrf2/Keap-1 Pathway

**Figure 6:**
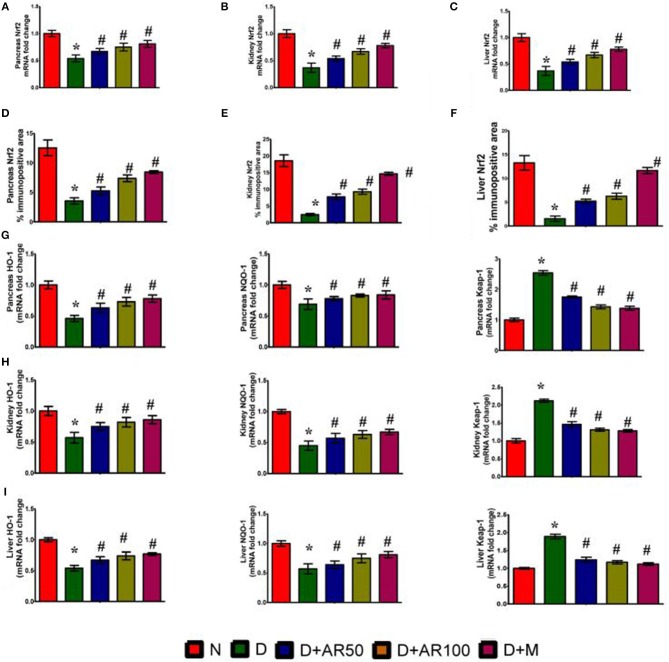
Effect of *Annona reticulata* L on **(A)** Pancreas Nrf2 mRNA fold changes **(B)** Kidney Nrf2 mRNA fold changes **(C)** Liver Nrf2 mRNA fold changes **(D)** Pancreas Nrf-2% immunopositive area **(E)** Kidney Nrf-2% immunopositive area **(F)** Liver Nrf-2% immunopositive area **(G)** Pancreas HO-1, NQO-1, and Keap-1 mRNA fold changes **(H)** Kidney HO-1, NQO-1, and Keap-1 mRNA fold changes and **(I)** Liver HO-1, NQO-1, and Keap-1 mRNA fold changes. Each value is mean ± SD of six rats in each group.^*^significant compared to normal control group *p* < 0.01. ^#^significant compared to diabetic group *p* < 0.01.

Figure 6 illustrates the Nrf2 mRNA expression levels, Nrf2% immunopositive area, HO-1 mRNA expression levels, NQO-1 mRNA expression levels and Keap-1 mRNA expression levels in pancreas, kidney, and liver. The diabetic rats showed down-regulated expression in the mRNA expression levels of Nrf2 (Figures 6A–C), with increased Nrf2 immunopositive area (Figures 6D–F) in pancreas, kidney and liver. Furthermore, diabetic rats presented with down-regulated mRNA expression levels of HO-1, NQO-1 with an up-regulated expression of Kelch-like ECH associated protein 1 (Keap-1) mRNA expression levels in pancreas (Figure 6G), kidney (Figure 6H), and liver (Figure 6I). Treatment of diabetic rats with 50 or 100 mg/kg *A. reticulata* ethanolic seed extract and metformin for 42 days significantly elevated the mRNA expression levels of Nrf2, HO-1, NQO-1 with reduced Keap1 mRNA expression and Nrf2 immunopositive area in the pancreas, kidney, and liver.

### Effect of *A. reticulata* on Anti-oxidant Enzymes

**Figure 7:**
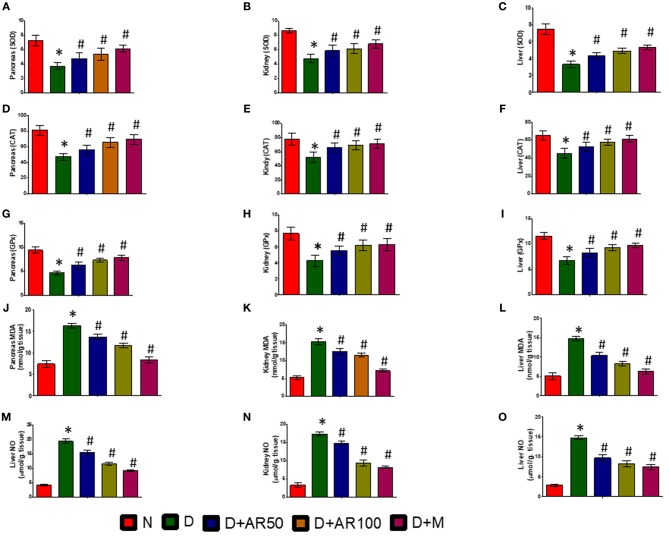
Effect of *Annona reticulata* L on **(A)** Pancreas SOD **(B)** Kidney SOD **(C)** Liver SOD **(D)** Pancreas CAT **(E)** Kidney CAT **(F)** Liver CAT **(G)** Pancreas GPx **(H)** Kidney GPx **(I)** Liver GPx **(J)** Pancreas MDA **(K)** Kidney MDA **(L)** Liver MDA **(M)** Pancreas NO **(N)** Kidney NO **(O)** Liver NO. Each value is mean ± SD of six rats in each group.^*^significant compared to normal control group *p* < 0.01. ^#^significant compared to diabetic group *p* < 0.01.

Figure 7 depicts activities of enzymatic antioxidants, such as SOD (Figures 7A–C), CAT (Figures 7D–F), and GPx (Figures 7G–I), along with the levels of MDA (Figures 7J–L) and NO (Figures 7M–O) in pancreas, kidney, and liver of control and experimental rats. The results revealed significantly increased levels of MDA and NO and significantly decreased activities of SOD, CAT, and GPx in pancreas, kidney, and liver of diabetic rats. However, treatment of diabetic rats with 50 or 100 mg/kg *A. reticulata* ethanolic seed extract or metformin for 42 days significantly improved the oxidative status by means of inducing a significant decrease in levels of MDA and NO and a significant increase in activities of SOD, CAT, and GPx in pancreas, kidney, and liver.

### Effect of *A. reticulata* on Pro-Inflammatory Parameters

**Figure 8:**
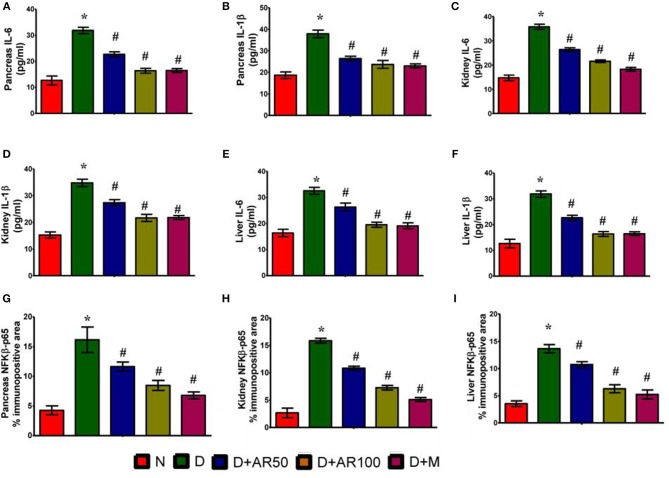
Effect of *Annona reticulata* L on **(A)** ELISA pancreatic IL-6 levels **(B)** ELISA pancreatic IL-1β levels **(C)** ELISA kidney IL-6 levels **(D)** ELISA kidney IL-1β levels **(E)** ELISA liver IL-6 levels **(F)** ELISA liver IL-1β levels **(G)** Pancreas NF-Kβ p65% immunopositive area **(H)** Kidney NF-Kβ p65% immunopositive area **(I)** Liver NF-Kβ p65% immunopositive area.

ELISA results demonstrated the inflammatory reaction in pancreas (Figures 8A,B), kidney (Figures 8C,D), and liver (Figures 8E,F) of diabetic rats, as evidenced by increased levels of inflammatory cytokines IL-6 and IL-1β. Further, the immunohistochemistry results depicted an increase in expression of pancreatic, kidney, and liver NF-κB p65 in diabetic rats, when compared with that of the same expression in control rats (Figures 8G–I). On the other hand, diabetic rats administered with either 50 or 100 mg/kg *A. reticulata* ethanolic seed extract and metformin for 42 days resulted in reduced IL-6, IL-1β, and NF-κB p65 expression in pancreas, kidney, and liver, in comparison with IL-6, IL-1β, and NF-κB p65 expression in diabetic rats.

## Discussion

Diabetes mellitus is a metabolic disorder characterized by the presence of high levels of glucose in blood that occurs either due to insulin's deficiency or malfunction (41). In recent years, the prevalence of diabetes has been increasing at an alarming rate and is the reason for uncountable human misery and millions of deaths worldwide. This dramatic increase in prevalence of diabetes warrants an urgent need to search for effective treatment strategies. Although a wide range of glucose lowering drugs are currently available in the market, they have not gained much significance due to drug resistance, side effects and toxicity. Now-a-days, usage of plant-based drugs is gaining importance because of its safe to use and non-toxic nature. At this juncture, the present study was carried out to assess the possible protective effect of 50 or 100 mg/kg body weight ethanolic seed extract of *A. reticulata* on type 2 diabetes rat model. Type 2 diabetes was induced by intraperitoneal injections of STZ and nicotinamide. In this study, one group of rats was also treated with metformin, the first line treatment option to manage type 2 diabetes. The results revealed that administration of either 50 or 100 mg/kg ethanolic seed extract of *A. reticulata* is effective in reducing DM complications through its anti-diabetic, antioxidant, anti-hyperlipidemic, and anti-inflammatory properties.

The results showed a significant weight loss with a significant increase in food and water consumption in STZ-induced diabetic rats, which are mainly owing to high wasting of muscles, rather than utilizing glucose as an energy source (42). The results are in consonance with the earlier findings of Guo et al. (43). Treatment of diabetic rats with *A. reticulata* extract or metformin significantly increased the body weight with a concomitant decrease in food intake and water consumption. This may be because of the defending nature of the *A. reticulata* extract or metformin on glucose metabolism by improving glucose utilization in insulin target tissues and by decreasing the activities of the gluconeogenic enzymes, thereby preventing muscle wasting.

Persistent hyperglycaemia, along with impaired glucose tolerance, higher hemoglobin glycation, reduced glycolysis and increased gluconeogenesis are some of the characteristic features of diabetes mellitus. In this study, constant hyperglycemia, impaired glucose tolerance, declined serum insulin levels and a significant elevation in HbA1c and HOMA-IR were observed in diabetic rats. A large body of literature also supported the present findings of this study (44–46). Treatment of diabetic rats with the 50 or 100 mg/kg *A*. *reticulata* ethanolic seed extract or metformin significantly reduced the blood sugar concentrations, with a rise in insulin levels and improvement in glucose tolerance, HbA1c, HOMA-IR, and insulin sensitivity. The data was also supported by the findings of histopathological studies where *A*. *reticulata* seed extract treatment to diabetic rats resulted in a significant increase in islet area and insulin positive cells. These results indicate the shielding effect of *A. reticulata* extract on the β-cells of the pancreas through its anti-diabetic potential and thereby restores the normal functioning of cells (47). The effects of 100 mg/kg *A. reticulata* seed extract and metformin are almost similar with insulin levels being higher in *A. reticulata* seed extract treated rats compared to metformin treated rats. The improvement in these metabolic alterations proved the effectiveness of *A. reticulata* seed extract in combating diabetic complications through its anti-diabetic nature.

Hyperlipidemia, a potential risk factor for cardiovascular disease, is a common complication associated with type 2 diabetes condition (48). Under normal physiological conditions, insulin activates lipoprotein lipase, which acts on triglycerides to separate fatty acids and glycerol. These fatty acids then undergo either oxidation to generate energy or re-esterification to store it in body tissues (49). Under diabetic condition, insulin resistance and/or deficiency inactivate lipoprotein lipase, which leads to a condition of a hyperglyceridemia. A normal biological level of LDL plays a role in the transportation of cholesterol from liver to other tissues of the body, whereas in contrast, HDL transports endogenous cholesterol from body tissues to liver where they get metabolized and excreted. Increased levels of LDL, which deposits cholesterol in arteries, leads to coronary heart disease (50), while high levels of HDL avoids atherosclerosis by preventing cholesterol deposition (51). In this study, the induced type 2 diabetic condition in rats resulted in an increased level of total cholesterol, triglycerides, LDL, in association with reduced levels of HDL. The observed findings are consistent with other published reports (52, 53). The administration of 50 or 100 *A. reticulata* seed extract or metformin to diabetic rats markedly improved hyperlipidemic condition in rats by either improved secretion of insulin and/or enhanced insulin sensitivity.

The assessment of kidney functional markers gives valuable information regarding the functionality of kidneys. In this present investigation, the serum levels of the BUN, creatinine levels, uric acid, urine volume, and urinary protein were significantly elevated with a significant decline in CCr, indicating the deviation from normal kidney functioning to eliminate waste from kidneys in diabetic rats. The deteriorated alterations in kidney parameters, such as kidney index, diameters of renal corpuscles, glomerulus and Bowman's capsule, in association with distorted glomerular morphology, are an indication of kidney damage in diabetic rats. The results supported the findings of Giribabu et al. (15) who reported deteriorated alterations in kidney functional markers and histopathological observations in diabetic rats. However, 50 or 100 mg/kg *A. reticulata* or metformin treatment to diabetic rats markedly improved the kidney functions as evidenced by reversal of all kidney functionality markers, demonstrating the renoprotective of *A. reticulata* ethanolic seed extract.

The hepatic damage in the diabetic rats was evident from increased levels of liver enzymes, such as ALT, AST, ALP, ACP, and GGT in serum. The augmented levels of these marker enzymes are an indicator of hepatocellular damage, which allows these liver functional enzymes to escape from cytosol into the bloodstream (54). The present observations corroborate with the earlier findings of an increased serum levels of ALT, AST, ALP, ACP, and GGT in diabetic rats (55). The alterations in these marker enzymes were supported by deranged liver functional markers, such as steatosis, hepatocyte ballooning and necroinflammation, along with an increased liver index and decreased glycogen content in diabetic rats. Further, liver damage was evidenced by histopathological studies where distorted morphology with hepatocellular necrosis and vacuolization were observed in diabetic rats. The structural alterations in the present study might be due to observed alterations in liver markers (56). While, diabetic rats, treated with either 50 or 100 mg/kg *A. reticulata* and metformin, significantly prevented the histopathological changes and restored the liver enzymatic and functional markers, suggesting the role of *A. reticulata* seed extract in restoring the liver damage caused by diabetes.

The oxidative stress induced by reactive oxygen species is considered to be a common pathophysiology in diabetes-mediated complications (57). Oxidative stress in cells overwhelms when there is a loss of balance between oxidants and antioxidant defenses (58). In this study, the markers of oxidative status, such as MDA levels (measure of lipid peroxidation) and NO levels, were significantly elevated with a significant diminution in activities of SOD, CAT, and GPx in pancreas, kidney, and liver of diabetic rats. The present findings are in par with earlier observations (59). While the administration of 50 or 100 mg/kg ethanolic seed extract of *A. reticulata* to diabetic rats caused a significant improvement in all five markers of oxidative stress, suggesting the anti-oxidant effect of *A. reticulata* seed extract on diabetes-induced oxidative stress. Regulation of cellular redox homeostasis is mainly governed by an endogenous anti-oxidative stress pathway, the Nrf2/Keap1/ARE pathway. Under normal biological conditions, cytoplasmic Nrf2 binds to Keap1, which restrict its access to nucleus to bind ARE, in order to transcriptionally activate Nrf2-target genes such as NQO1 and HO-1 (60). In the current study, diabetic rats have shown significantly down-regulated mRNA expression of Nrf2, NQO1, and HO-1, with a significant up-regulation in the Keap1 mRNA expression and Nrf2 protein expression in pancreas, kidney and liver. While 50 or 100 mg/kg ethanolic seed extract of *A. reticulata* to diabetic rats significantly attenuated all these gene and protein expressions to protect oxidative damage in pancreas, kidney, and liver through its anti-oxidant potential (60). The anti-oxidant effect of *A. reticulata* is almost similar to the effect observed by metformin.

Earlier research has pointed out the involvement of DM-induced oxidative stress in the activation of NF-kβ (61). NF-kβ is known to activate pro-inflammatory cytokines, which are considered to be important in an inflammatory reaction. In this study, an up-regulated expression of NF-κβ p65, along with elevated levels of NF-κβ p65, IL-6, and IL-1β in pancreas, kidney, and liver, were observed in diabetic rats. The results are in par with earlier observations (62, 63). After treatment of diabetic rats with 50 or 100 mg/kg *A. reticulata* ethanolic seed extract or metformin has resulted in a significant improvement in levels and expression of these inflammatory markers, suggesting the anti-inflammatory effect of *A. reticulata* ethanolic seed extract to protect diabetes-mediated complications.

Inflammation has a vital role in the progression of DM and is therefore related to increased insulin resistance and decreased response in insulin target tissues (64). NF-kβ and TNF-α are critical mediators of insulin resistance and beta cells dysfunction in the pancreas, contributing to the development of diabetes mellitus, and also anomaly in lipid metabolism (65). Persistent hyperglycaemia results in increased production of free radicals and other inflammatory cytokines, which activate NF-kβ (66). In normal conditions, NF-kβ is known to exist in an inactive state in the cytoplasm and then binds via p50 and p65 units to Ikβ, which then acts as an inhibitory protein (67). Upon stimulation, it becomes separated from Ikβ, and the active p65-NF-kβ translocates to the nucleus where it binds and activates the expression of pro-inflammatory cytokines (68, 69). Therefore, measurement of the accessibility of active p65-NF-kβ will serve as an important factor indicating the translocation of NF-kβ to the nucleus. Increased levels of the active form of NF-kβ, along with an increased level of inflammatory cytokines, were observed in liver, kidney and pancreatic tissues. Treatment of the diabetic animals with the *A. reticulata* seed extract resulted in a significant reduction in inflammatory cytokines, thereby increasing the insulin sensitivity in diabetic rats, suggesting a putative role of seed extracts of *A. reticulata* in alleviating diabetes mellitus through its anti-inflammatory effect.

In conclusion, the treatment of *A. reticulata* seed extract to diabetic rats significantly improved the metabolic alterations, dyslipidemia, oxidative, and inflammatory status through its anti-diabetic, anti-hyperlipidemic, anti-oxidative, and anti-inflammatory effects. Therefore, the seed extract of *A. reticulata* could be as an ideal treatment option for diabetes mellitus.

## Ethics Statement

This study was carried out in accordance with the recommendations of ethical guidelines provided by the Animals Ethics Committee of Changzhi Medical College. The protocol was approved by the institute's animal ethical committee following the guidelines of National Institute of Health.

## Author Contributions

WW: data acquisition and manuscript preparation. YL: data acquisition and analysis. ZT: experimental design and manuscript preparation.

### Conflict of Interest

YL was employed by Innoscience Research Sdn Bhd. The remaining authors declare that the research was conducted in the absence of any commercial or financial relationships that could be construed as a potential conflict of interest.

## Supplementary Material

Supplementary Figure 1 Effect of *Annona reticulata* L. on **(A)** HbA1c **(B)** Islets area/total pancreatic area (%). **(C)** Insulin % immunopositive area. **(D)** Immunohistochemistry of insulin in pancreas. Each value is mean ± SD of 6 rats in each group. ^*^Significant compared to normal control group *p* < 0.01. ^#^Significant compared to diabetic group *p* < 0.01.

Supplementary Figure 2 Effect of *Annona reticulata* L. on **(A)** Immunohistochemistry of pancreatic Nrf2. **(B)** Immunohistochemistry of kidney Nrf.2 **(C)** Immunohistochemistry of liver Nrf2. Each value is mean ± SD of 6 rats in each group. ^*^Significant compared to normal control group *p* < 0.01. ^#^Significant compared to diabetic group *p* < 0.01.

Supplementary Figure 3 Effect of *Annona reticulata* L. on **(A)** Immunohistochemistry of pancreatic NF-Kβ p65. **(B)** Immunohistochemistry of kidney NF-Kβ p65. **(C)** Immunohistochemistry of liver NF-Kβ p65. Each value is mean ± SD of 6 rats in each group. ^*^Significant compared to normal control group *p* < 0.01. ^#^Significant compared to diabetic group *p* < 0.01.

Supplementary Figure 4 Effect of *Annona reticulata* L. on **(A)** ELISA pancreatic NF-Kβ p65 levels. **(B)** ELISA kidney NF-Kβ p65 levels. **(C)** ELISA liver NF-Kβ p65 levels. Each value is mean ± SD of 6 rats in each group. ^*^Significant compared to normal control group *p* < 0.01. ^#^Significant compared to diabetic group *p* < 0.01.
