# Supplementary figures and images for: Antidiabetic, Antihyperlipidemic, Antioxidant, Anti-inflammatory Activities of Ethanolic Seed Extract of Annona reticulata L. in Streptozotocin Induced Diabetic Rats
Source: Front Endocrinol (Lausanne). 2019 Oct 23;10:716. doi: 10.3389/fendo.2019.00716 (PMC6819323; doi:10.3389/fendo.2019.00716)

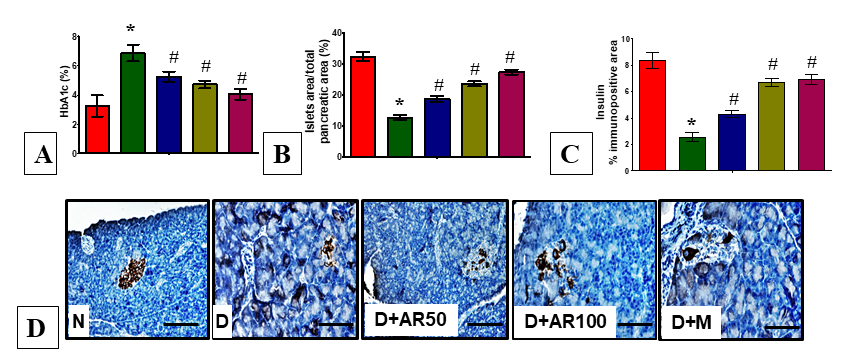

Supplement: Supplementary Figure 1 — Effect of Annona reticulata L. on (A) HbA1c (B) Islets area/total pancreatic area (%). (C) Insulin % immunopositive area. (D) Immunohistochemistry of insulin in pancreas. Each value is mean ± SD of 6 rats in each group. *Significant compared to normal control group p < 0.01. #Significant compared to diabetic group p < 0.01. [file Image_1.TIF]

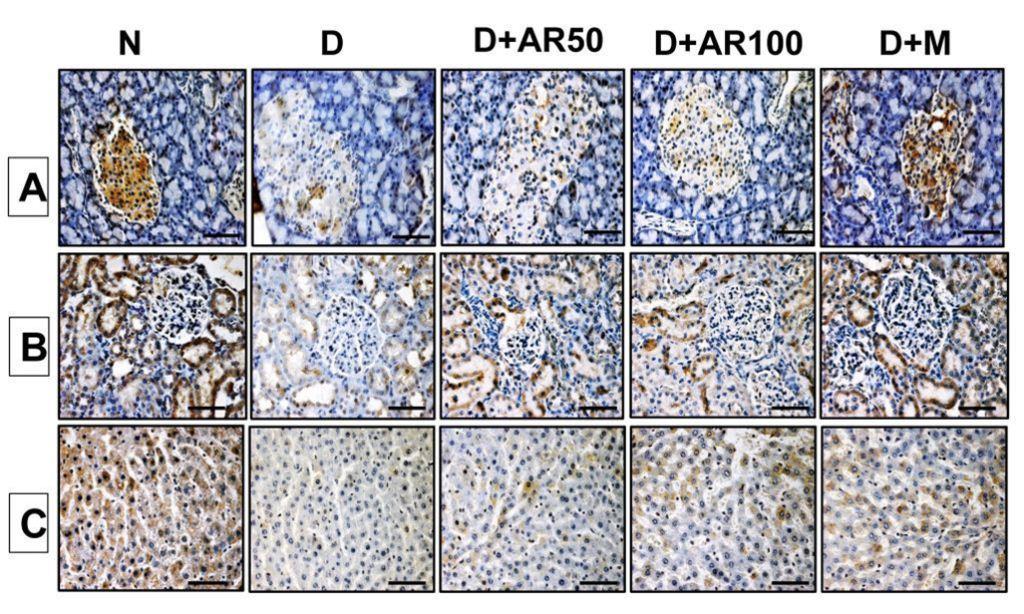

Supplement: Supplementary Figure 2 — Effect of Annona reticulata L. on (A) Immunohistochemistry of pancreatic Nrf2. (B) Immunohistochemistry of kidney Nrf.2 (C) Immunohistochemistry of liver Nrf2. Each value is mean ± SD of 6 rats in each group. *Significant compared to normal control group p < 0.01. #Significant compared to diabetic group p < 0.01. [file Image_2.JPEG]

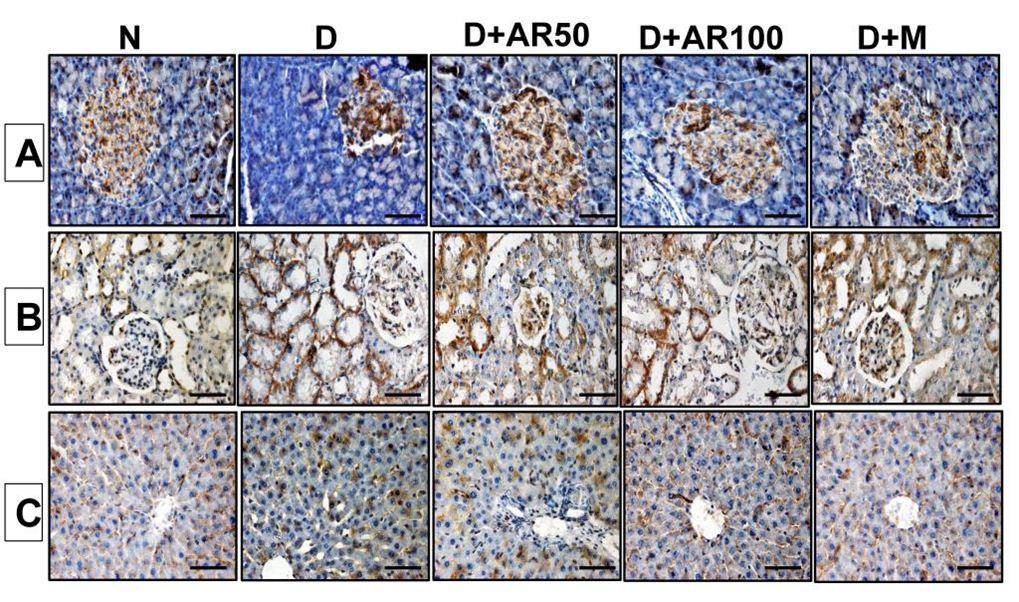

Supplement: Supplementary Figure 3 — Effect of Annona reticulata L. on (A) Immunohistochemistry of pancreatic NF-Kβ p65. (B) Immunohistochemistry of kidney NF-Kβ p65. (C) Immunohistochemistry of liver NF-Kβ p65. Each value is mean ± SD of 6 rats in each group. *Significant compared to normal control group p < 0.01. #Significant compared to diabetic group p < 0.01. [file Image_3.JPEG]

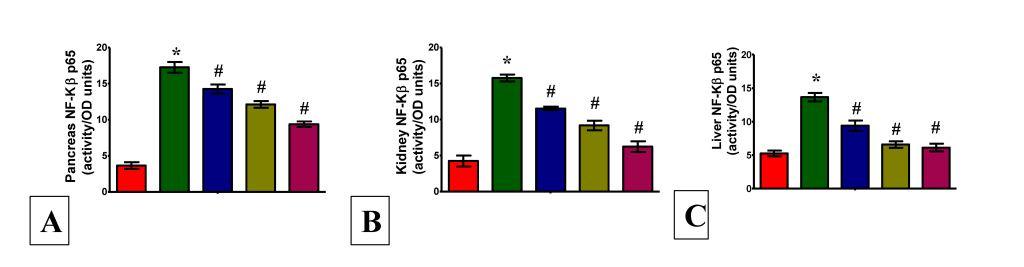

Supplement: Supplementary Figure 4 — Effect of Annona reticulata L. on (A) ELISA pancreatic NF-Kβ p65 levels. (B) ELISA kidney NF-Kβ p65 levels. (C) ELISA liver NF-Kβ p65 levels. Each value is mean ± SD of 6 rats in each group. *Significant compared to normal control group p < 0.01. #Significant compared to diabetic group p < 0.01. [file Image_4.JPEG]
